# Analysis of the Similarity between Injection Molding Simulation and Experiment

**DOI:** 10.3390/polym16091265

**Published:** 2024-05-01

**Authors:** Julia Knoll, Hans-Peter Heim

**Affiliations:** Institute of Material Engineering, Polymer Engineering, University of Kassel, 34125 Kassel, Germany; heim@uni-kassel.de

**Keywords:** injection molding, injection molding flow simulation, Moldflow^®^, similarity analysis

## Abstract

In the plastics industry, CFD simulation has been used for many years to support mold design. However, using simulation as a substitute for experimentation remains a major challenge to this day. This is due to the unknown congruence between simulation and experiment. The present work focuses on a comparison between simulation (generated with the software Moldflow Insight Ultimate from Autodesk Inc., San Francisco, CA, USA) and experiment by using molds of different complexity, where, in contrast to a large number of previous investigations, both the characteristics of the parts and the time series of the process parameters were compared with each other. For this purpose, the high-resolution time series of the process parameters injection pressure, flow rate, and cavity pressure as well as the mass and the dimensions of the manufactured parts were acquired during the experiments and the results were compared with the computations obtained from the simulation. In addition, potential causes like the material data, mesh and solver parameter, and the machine-specific behavior were analyzed to assess which of these causes may be decisive for a deviation between simulation and experiment.

## 1. Introduction

The injection molding process is a manufacturing process that enables the production of directly usable parts in short cycle times. The cavities of the injection mold specify the shape of the polymer parts to be manufactured. In order to design the molds and the corresponding cavities, the mold shape is designed, constructed and mechanically manufactured as part of the developing process. To avoid potential defects (such as weld lines, sink marks, short shots, or ejection problems) at an early stage of mold development, commercial simulation software can be used [1,2,3,4]. This software maps the flow, cooling, and shrinkage behavior of the melt in the cavity and thus provides essential information that can be used for designing the injection mold. The findings are subsequently employed to make adjustments to the mold such as the cavity, the gating system, the cooling channels, or the mold venting, among others. However, the operating point of the machine cannot be derived from this. This is due to the fact that the machine operator does not know whether the simulation has a high similarity to the experiment and can be used as a substitute for an experiment.

### 1.1. Simulation of the Injection Molding Process Based on Computational Fluid Dynamics (CFD)

Various commercial simulation programs are available for simulating the injection molding process, such as Moldflow from Autodesk Inc., Moldex 3D from CoreTech Systems Co., Ltd. (Zhubei City, Taiwan) or Sigmasoft from Sigma Engineering GmbH (Aachen, Germany).

**Figure 1:**
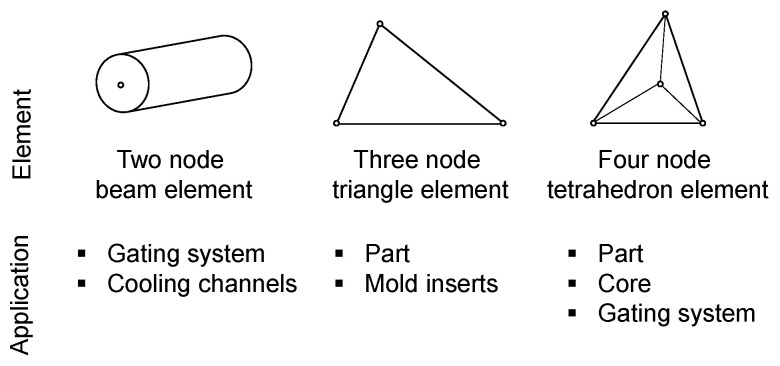
Element types for meshing different geometries.

At the beginning of each simulation study, the CAD file of the part needs to be imported in the software. Optionally, cooling channels, the mold model, the gating system, and a hot runner model can also be added. To perform an analysis, the geometry of the part or the volume flowed through must then be divided into finite sub-regions. To discretize the geometry, a finite element mesh is generated. This mesh can be created from three different element types, which in turn are defined by nodes, i.e., coordinates in space. The element types are shown in Figure 1.

**Figure 2:**
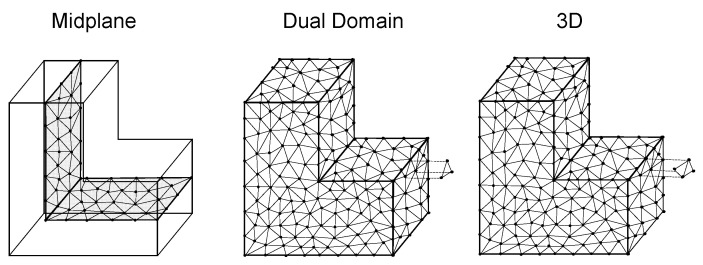
Mesh types for discretizing the geometry.

In Moldflow, the midplane, dual domain, and three-dimensional (3D) meshes are available for discretizing the geometry (cf. Figure 2). In both the midplane and dual domain meshes the midplane or surface is represented by triangular elements. In the case of the dual domain mesh, the thickness of the part is also calculated using the distance between the opposite surfaces. To also mesh the volume in the thickness direction, the complete geometry of the three-dimensional mesh (3D mesh) is meshed using tetrahedral elements. To parameterize the mesh fineness, the global edge length (GEL) is used, which specifies the average edge length of the triangle or tetrahedron elements. For 3D meshes, the number of layers across thickness (NLT) is additionally defined to parameterize the mesh fineness in thickness direction. In [5] the advantages and disadvantages of the different mesh types are presented.

Depending on the mesh type, different assumptions are made to calculate the flow. For the midplane and dual domain mesh, the calculation is based on the generalized Hele-Shaw flow model, with the following assumptions [5,6]:

- Laminar flow of a Newtonian fluid;
- Neglect of inertia and gravity effects;
- Filling of the cavity is considered as a 2D problem, assuming symmetry at the midplane;
- Neglect of wall slip;
- In-plane heat conduction is negligible compared to heat conduction in thickness direction;
- Neglect of heat convection in thickness direction;
- Neglect of heat losses at the edges of the triangular elements.

Due to the simplifications, the Hele-Shaw models cannot represent the flow behavior of the melt at the flow front, the flow behavior near and at the solid wall, the merging of two or more fluid flows, and the kinematics in the areas where shear and elongation deformation cause significant stresses (sprue, ribs, abrupt thickness changes) [5]. As a further development, the 3D models were developed with the following assumptions [5,6]:

- Flow according to the Navier–Stokes equations;
- Calculation of pressure, temperature and the three velocity components at each node;
- Consideration of heat conduction in each direction;
- Optional: consideration of inertia and/or gravity effects.

The basic equations for the flow calculation in the filling and packing pressure phase are the continuity equation, the momentum equation, and the energy equation, which are described in [7,8]. The complete Navier–Stokes equations are presented in [1,9,10]. Variables to be determined thereby are the velocity vector, density, pressure, and temperature as a function of spatial coordinates and time. No analytical solutions are known for the equations (except for special cases such as a flat plate), so the systems of differential equations are solved numerically for technically relevant problems. For this purpose, the problem is discretized, i.e., the partial derivatives (differentials) are converted into finite differences. The differential equations can be solved numerically by providing flow variables such as density and velocity vectors at the nodes of the mesh. Here, the differential at one node can be replaced by the difference in the values at the neighboring nodes [9,10]. A large number of different methods exist for the numerical solution of the differential equations [11]. The challenges to solve the nonlinear systems of equations as well as a benchmark of the different methods are presented in [12].

Since the approximations of the Hele-Shaw flow models, as used in midplane and dual domain meshing, can lead to significant errors due to the loss of geometric information as well as the simplification of the flow properties in the thickness direction [13], only 3D meshing, which is also predominantly used in industry, will be considered in this work. Thereby, the simplifications made depending on the mesh type used are stored in the software and consequently identical for each simulation depending on the mesh type. In contrast, the mesh and solver parameters are usually set intuitively by the operator. To what extent the mesh fineness and the solver parameters have an influence on the calculation result depends on the use case and is therefore unknown to the user.

To model the viscosity of the polymer melt, in most commercial simulation programs the Cross-Williams, Landel, and Ferry (Cross-WLF) model is used, which describes the viscosity η as a function of the shear rate $\dot{\gamma }$, pressure p, temperature T, zero shear viscosity $\eta_{0}$, critical stress level at transition to shear thinning $\tau^{*}$, the power law index slope in shear thinning domain n and the glass transition temperature $T^{*}$ [14,15,16,17]:

$$\eta =\frac{\eta_{0}}{1+{\left(\frac{\eta_{0}\dot{\gamma }}{\tau^{*}}\right)}^{1-n}}\;\mathrm{with}\;\eta_{0}=D_{1}exp\left[-\frac{A_{1}(T-T^{*})}{A_{2}+(T-T^{*})}\right],\;A_{2}={\tilde{A}}_{2},+D_{3}p\;\mathrm{and}\;T^{*}=D_{2}+D_{3}p$$

The coefficients n, $\tau^{*}$, $A_{1}$, ${\tilde{A}}_{2}$, $D_{1}$, $D_{2}$, $D_{3}$ are determined using curve fitting based on measured viscosity data. To determine the specific volume v of the polymer as a function of the temperature T and pressure p, the 2-domain Tait pvT model, which is given by the following equation, is used:

$$v\left(T,p\right)=v_{0}\left(T\right)\left[1-C\ln\left(1+\frac{p}{B\left(T\right)}\right)\right]+v_{t}\left(T,p\right)$$

In the equation, $v_{0}\left(T\right)$ is the specific volume at zero gauge pressure, C is a constant with a value of 0.0894, B accounts for the pressure sensitivity of the material and the term $v_{t}\left(T,p\right)$ accounts for the volume decrease due to crystallization. Above and below the transition temperature, the equations for $v_{0}\left(T\right)$, $B\left(T\right)$ and $v_{t}\left(T,p\right)$ differ. The complete equations are presented in [18,19].

A large number of investigations have already been carried out, e.g., to determine the influence of the cooling medium on warpage [20], to analyze the impact of parameter changes on the weight [21], to improve warpage analysis [22], and to analyze the impact of the kind of mesh on the temperature and pressure [23] based on simulation studies. In order to use the information for the actual process, a comparison must be made with experimental data.

### 1.2. Comparison of Simulation and Experiment

Various studies have already compared the results of numerical computations with data measured during the experiments. It has been shown that the model results (here: maximum injection pressures, cooling calculations, shrinkage, and warpage) are in some cases underestimated and in other cases overestimated depending on the material or model used (here: dual domain and 3D calculations) compared with the experimentally measured values [24]. Clear correlations between the model or material used and the congruence between simulation and experiment could not be observed. In [25] it was already shown that the calculated and actual filling behavior, specifically the filling time, can deviate from each other as a result of machine-specific influencing variables. However, simulation and experiment show differences not only with regard to the process parameters but also with regard to the properties of the injection-molded parts. Comparisons of the calculated mass and dimensions of the parts with experimental measurements [26,27,28] have already shown that the calculated and experimentally measured part characteristics can deviate from each other when the machine settings (mold temperature, melt temperature, cooling time, packing pressure time, packing pressure, and filling time) were varied. It was shown that the influence of various parameter changes can be qualitatively represented using computational results compared to the experiment, but not quantitatively. According to Tercan et al. [27], the offset between simulation and experiment illustrates that individual effects in the process are under- or overestimated by the simulation.

In addition to process parameters and general part characteristics (such as mass), the focus in recent years has increasingly been on calculating the structural properties of injection molded parts using simulation software. In this context, the ability of simulation software to model reinforced or foamed polymers has been investigated. For both glass fiber-reinforced [29] and polymers filled with flexible wires [30], it was found that no model exists to date that comprehensively maps the orientations of the fibers or wires. Various models also exist for calculating foamed polymers, e.g., to calculate the cell diameter and local density, whereby these also partly show significant deviations from the actual cell dimensions [31]. In contrast, studies exist that demonstrate a high level of similarity between simulation and experiment, e.g., in [32], where the focus was on the alignment of component stresses. The investigations show that commercially available simulation programs are not yet completely able to calculate the properties of injection-molded parts. The presented literature already indicates that the correspondence between simulation and experiment depends on the part characteristics considered and the respective production scenario.

### 1.3. Objective of the Investigations

As part of the digitalization of injection molding processes, the aim is often to substitute experiments, which are usually associated with high costs, with a digital representation. Commercially available simulation programs are therefore used to obtain information about the process as early as possible in the development process of injection molds. However, the extent to which simulations can actually be used as a substitute for experiments is still unknown. On the one hand, it remains to be investigated to what extent simulation and experiment deviate from each other depending on the production scenario depicted. A comprehensive assessment of this has not yet been published in the literature. As a distinction to previous investigations, not only the properties of the final parts were compared as part of the studies, but also the time series of the process parameters, which provide more detailed information on the process sequence. On the other hand, potential causes that result in a deviation between the measured and calculated values were discussed and analyzed in detail. In this context, the boundaries of the simulation, the correspondence of the material data and the influence of the mesh and solver parameters were considered.

For the investigations, different process conditions were achieved by varying the operating points in both the simulation and the experiment, and the time series of the process parameters as well as the part characteristics were measured during the experiment and contrasted with the simulation results.

## 2. Materials and Methods

**Figure 3:**
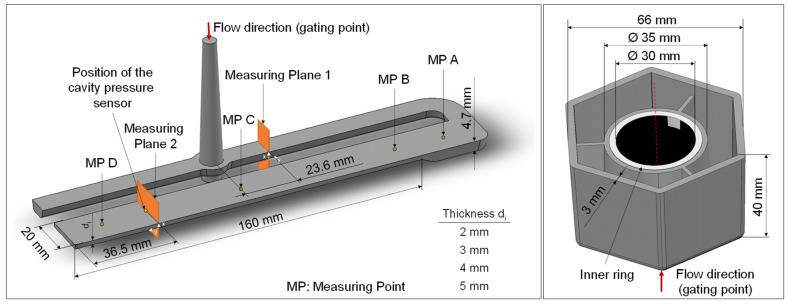
Specimens investigated, **left**: flat bar with a varying thickness, **right**: hexagonal shaped part with a mutable inner ring (light gray).

To cover various production scenarios four different variants of the flat bar (Figure 3, left side) and two different variants of the hexagonal shaped part (Figure 3, right side) were investigated. The variations in the flat bar thicknesses were achieved by employing different mold inserts. The inserts used only impact the thickness of the 160 mm long test specimen, as the gate remains consistent across all specimen variants. The mold was equipped with a pressure sensor to measure the cavity pressure. The position of the sensor is shown in Figure 3. The two variants of the hexagonal shaped part differ in the inner ring (shown as a light grey ring in Figure 3). A core pull in the mold was used to produce the part with and without the inner ring. Both molds are single-cavity molds, whereby the second cavity was specifically closed in the case of the flat bar. The flat bar mold is a cold runner mold, as can be seen from the sprue. The hexagon mold, on the other hand, has a hot runner, resulting in parts without sprue.

Hydromechanical injection molding machine (IMM) with the type “Arburg Golden Edition 320 C” (A) of the company Arburg GmbH + Co KG (Loßburg, Germany) was used for the production of the flat bars. Due to the larger mold dimensions, the IMM “Arburg Allrounder 470 S” (B) from the same manufacturer was used for the production of the hexagonal shaped parts. Further information about the machines can be found in Table 1.

For the production of the flat bars and hexagonal shaped parts, the polyamide with the designation “Ultramid B3S” provided by the company BASF (Ludwigshafen am Rhein, Germany) was used. A description of the material properties can be found in the product datasheet [33]. Before commencing the injection molding experiments, the polyamide underwent a drying process for approximately 4 h at 80 °C using an air dryer (TORO-systems TR-Dry-Jet EASY 15, provided by GfK Gesellschaft für angewandte Kunststofftechnik, Igensdorf, Germany) until a moisture content of about 0.2% was attained. To simulate the experiment in an equivalent way, the material data characterized by the material manufacturer and stored in Moldflow were used to configure the simulation.

### 2.1. Description of the Experiments Using the Flat Bar (Use Case 1)

The experimental design for performing the simulations and experiments is shown in Table A2 in the Appendix A. The designation of the experimental points depends on the part thickness investigated (see Appendix A Table A3). According to the material characteristics, cylinder temperatures in the range of 250 °C and 270 °C were configured. The cylinder temperature T_c_ corresponds to the nozzle temperature in the experiment (with the corresponding heating zone temperatures shown in Table A1 in the Appendix A). The cylinder heating zone temperatures were controlled using the injection molding machine. T_m_, Q_inj_, and p_pack_ represent the mold temperature, flow rate, and packing pressure, respectively. As reference for T_m_, the return flow temperature of the coolant was selected, which was controlled in a closed circuit using an external cooling device. The values for Q_inj_ and p_pack_ were determined using a Latin Hypercube design, with flow rate intervals of (20 cm^3^/s, 60 cm^3^/s) and packing pressure intervals of (200 bar, 500 bar). The cylinder and mold temperatures were purposefully not determined according to the Latin Hypercube design since varying the temperatures from test point to test point would result in significantly increased experimental effort to ensure thermal equilibrium of the process. The transition point between flow rate-controlled injection and packing pressure was established through a filling study conducted during preliminary tests.

The packing pressure was specified using a profile comprising three phases. In the initial phase, there was a transition from injection pressure to packing pressure within a 0.05-s timeframe. The second phase involved maintaining the packing pressure for 15 s. The sole variable manipulated within the scope of the experimental design was the packing pressure level during this phase. Following this, the transition from packing pressure to 25 bar was executed within 0.05 s. The cooling time was 20 s for all experiments.

To ensure the reproducibility of the resulting part characteristics, three test specimens were manufactured for each parameter configuration. After adjustments of T_c_ and T_m_, approximately ten test specimens for each parameter configuration were manufactured before producing the test specimens. This was performed to establish thermal stability and ensure consistent processing conditions.

### 2.2. Description of the Experiments Using the Hexagonal Shaped Part (Use Case 2)

The experimental design for the configuration of the simulation and the experiment to manufacture the hexagonal shaped parts is shown in Table A4 in the Appendix A. Since the same material was used for the flat bar, the same cylinder and mold temperatures were chosen for the experimental design, which were also varied at fixed factor levels. The flow rate Q_inj_ and the packing pressure p_pack_ were varied within the intervals (20 cm^3^/s, 60 cm^3^/s) and (250 bar, 500 bar), respectively, according to the Latin Hypercube design. The transition points between flow-rate controlled injection and packing pressure were determined using a filling study during preliminary tests for both part variants. Thereby the dosing volumes were kept constant and the switchover volumes were adjusted to achieve 99% filling at the end of the filling phase for both part variants. The packing profile was identical to the packing profile of the flat bar, except that the packing time of the second phase was 24 s. The cooling time was 20 s for all experiments.

### 2.3. Acquisition of Part Characteristics and Process Parameters during the Experiments

To compare the properties of the parts computed in the simulation and those produced in the experiment various characteristics were used. For the flat bar, the part mass and the thickness were determined at four different measuring points (ct. Figure 3). The thicknesses of the flat bars produced were measured using a digital micrometer of the type “DIGI-MET 1865510” of the company Helios-Preisser (Gammertingen, Germany) with a resolution of 0.001 mm. The hexagonal shaped parts were measured with an image-based measuring system ”IM-7020” [34] provided by the company Keyence (Osaka, Japan), which was also used in the investigations in [35]. The measuring system is equipped with a movable measuring table with a double telecentric lens and a 6.6 mega pixel image sensor located above it. By placing the hexagonal shaped parts on their flat underside on the measuring table, images were taken using reflected light and the dimensions were measured.

The weighing was implemented using a precision scale with the designation “Practum 224-1S” supplied by the company Satorius AG (Göttingen, Germany). The flat bars were weighed with the sprue system, as mechanical removal would not be expedient and the masses of the parts in the simulation were also calculated including the sprue system. Since the hexagonal molded parts were produced with a hot runner, the parts were weighed without any sprue system. The dimensions and mass were consistently measured at uniform time intervals following the parts’ manufacturing process. These measurements were consistently conducted by the same individual to eliminate potential systematic errors during the assessment.

Continuous data recording during the entire experiments was implemented by using the OPC UA interfaces of the machines to obtain the time series of the injection pressure, flow rate, cavity pressure, and screw volume.

### 2.4. Simulation of the Injection Molding Processes

As CFD software, the commercial program Moldflow Insight Ultimate Version 2019.0.2 [36] was used. The settings of the simulation were chosen equivalent to the experimental designs shown in Table A2 and Table A4, with the mold temperature configured according to T_m_ and the temperature of the melt entering the mold according to T_c_. The machine, the material, and the mold steel were specified in Moldflow according to the materials used in the experiment. It should be noted that the plasticization of the material cannot be configured in the simulation, since the simulation only describes the process from the moment the melt enters the mold or, if included in the model, the hot runner.

**Figure 4:**
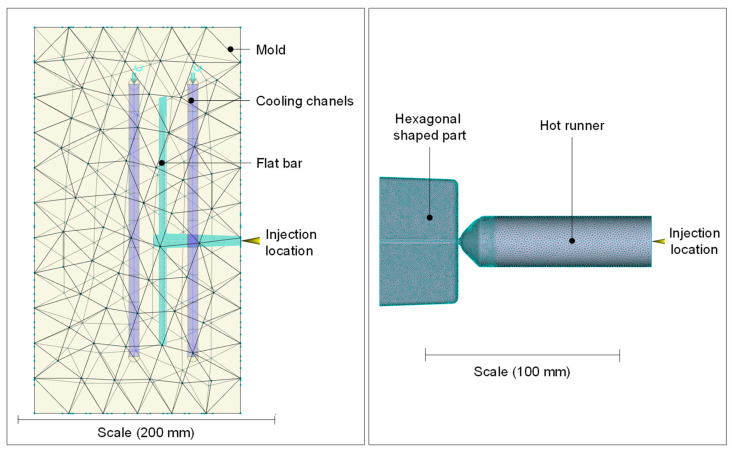
Simulation models of the flat bar (**left**) and hexagonal shaped part with hot runner (**right**).

The simulation models are visualized in Figure 4. As the injection molds were manufactured exactly according to the design dimensions using a high-precision CNC milling machine, the nominal design dimensions were used for the models. In preliminary investigations, the effects of the cooling channels and the mold model on the calculated part characteristics as well as the time series of the process parameters were investigated in detail. It was shown that the cooling channels as well as the mold model have no significant influence when simulating the hexagonal shaped part, whereas the computation times increased significantly. For this reason, the corresponding components were omitted in the simulation model of the hexagonal shaped part. It is crucial to underscore that this conclusion is specific to the mold model and the simulation results considered. The possibility of an influence on other simulation results or when employing different mold designs cannot be ruled out.

In addition to the experimental designs shown in the Appendix A, Table 2 lists the parameters that were used to configure the simulation studies.

To extract the data from the software for further data processing, the so-called analysis logs were exported, which contain the time series of the process parameters and the part masses. In addition, for describing the part dimensions and thicknesses, individual nodes of the mesh were selected in Moldflow at the corresponding measuring points (see Figure 3). By measuring the distance between the opposite nodes on the part surfaces, the thicknesses or rather dimensions after warpage were measured. In order to avoid mesh-related measurement differences, the model including the mesh was duplicated for each trial number of a part variant and only the simulation settings (which are described in Table A2 and Table A4) were adjusted according to the trial number.

## 3. Results and Discussion

### 3.1. Comparison of the Part Masses and Dimensions

For the evaluation of the ability of the simulation to calculate the part characteristics, the results of the flat bar (Use Case 1) are presented in Section 3.1.1 and those of the hexagonal part (Use Case 2) in Section 3.1.2. After a mere presentation of the results, the results of both Use Cases are discussed conclusively in Section 3.1.3.

#### 3.1.1. Use Case 1: Flat Bar

**Figure 5:**
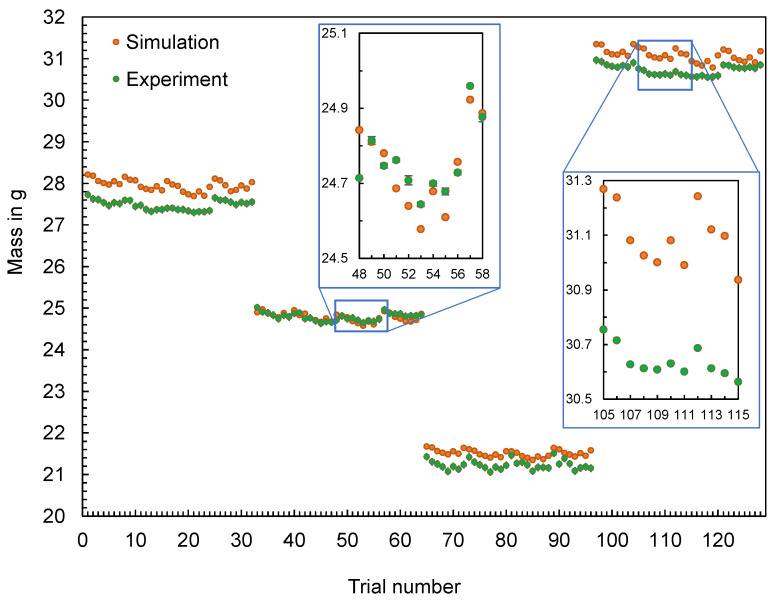
Computed and experimental measured masses of the flat bars with a thickness of 4 mm, 3 mm, 2 mm, and 5 mm (f.l.t.r.).

The calculated mass of the flat bars and the mass measured in the experiment are compared in Figure 5. The plot shows the mass depending on the order of the experiment, with samples of 4 mm thickness made first, followed by the 3 mm, 2 mm, and 5 mm thick flat bars.

**Figure 6:**
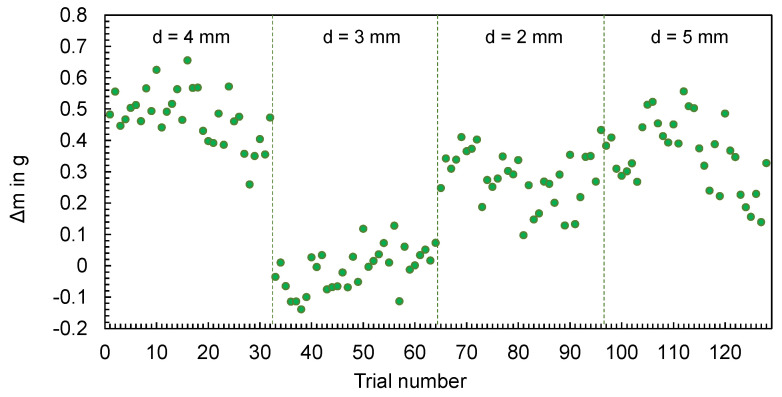
Deviation of the computed and mean measured masses of the flat bars.

In addition, Figure 6 illustrates the deviations between the calculated masses $m_{\mathrm{Sim}}$ and the mean values of the masses $m_{\mathrm{Exp},i}$ measured during the experiments, calculated according to Equation (1).

(1) $$∆m=m_{\mathrm{Sim}}-\sum_{i=1}^{i=n}\frac{m_{\mathrm{Exp},i}}{i}$$

The differences between the simulated and the experimentally measured mean masses are in the range between 0.0008 g and 0.6549 g. It can be seen that the difference varies depending on the geometry. In addition, the machine setting changes and their effect on the part mass (increase or decrease) can be mapped qualitatively for most parts, but not quantitatively. The effect of parameter changes on the part mass is thus better represented in contrast to the results in [21].

**Figure 7:**
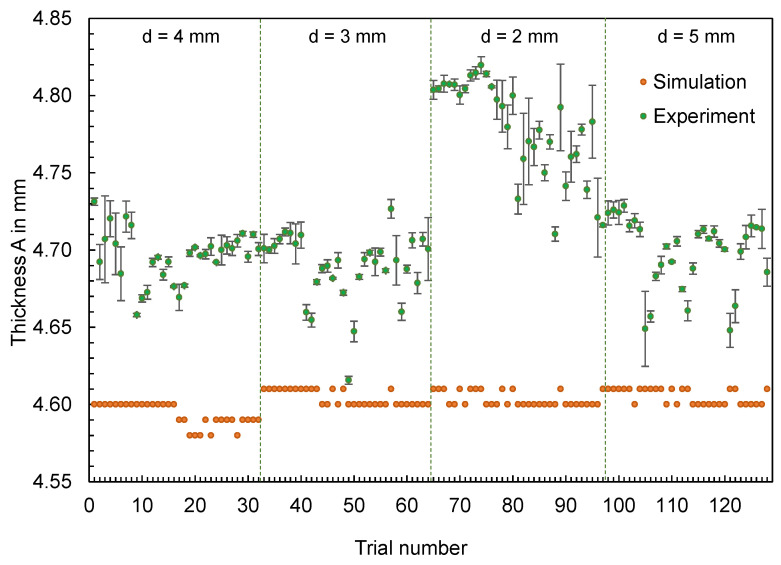
Computed and experimental measured thickness of the flat bars at measurement point A.

**Figure 8:**
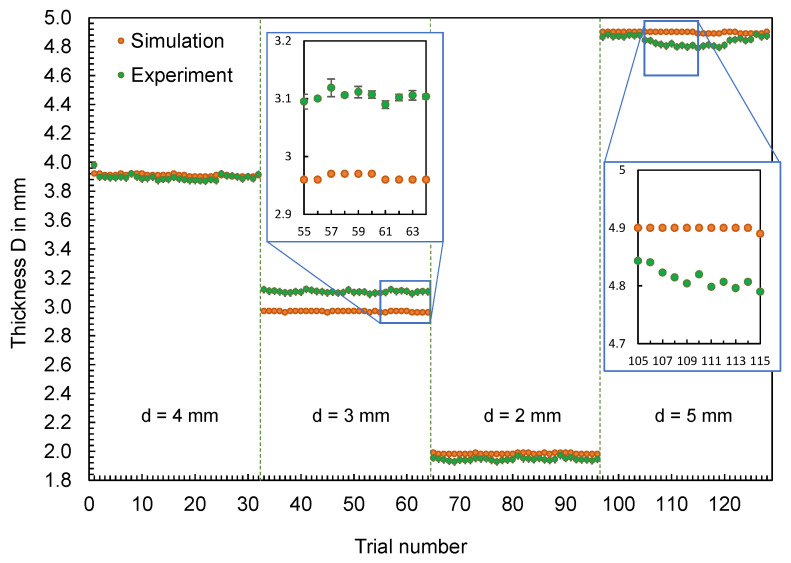
Computed and experimental measured thickness of the flat bars at measurement point D.

Supplementary to the part mass, the thicknesses of the flat bars were measured at four different measuring points (cf. Figure 3, left) in the experiment and were compared with the simulation results. The results for thicknesses A and D are shown in Figure 7 and Figure 8, respectively. The geometry of the mold inserts and the simulation models are identical at measuring point A for all part variants. In contrast, the geometries at measuring point D differ. Here, different mold inserts were used in the experiment to vary the thickness of the flat bars. The simulation models were modified according to the mold inserts used.

**Figure 9:**
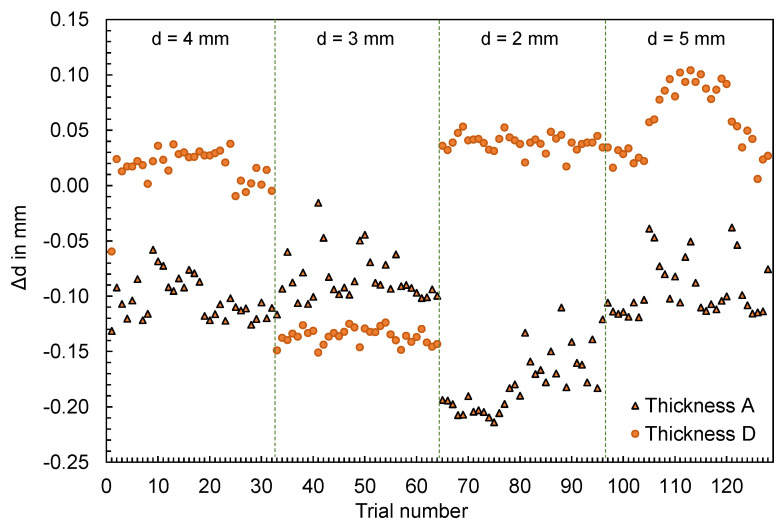
Deviation of the computed and mean measured thicknesses of the flat bars at measurement point A and D.

In addition to the results, Figure 9 visualizes the deviations of the thicknesses ∆d at the measuring points A and D depending on the trial numbers. The differences shown were determined according to Equation (2) from the calculated thicknesses $d_{\mathrm{Sim}}$ and the mean value of the measured thicknesses $d_{\mathrm{Exp},i}$.

(2) $$∆d=d_{\mathrm{Sim}}-\sum_{i=1}^{i=n}\frac{d_{\mathrm{Exp},i}}{i}$$

For both thickness A and thickness D, changes in thickness measured in the experiment, resulting from changes in the operating point, are not reproduced by the simulation. Depending on the geometry, a considerably different pattern can be seen. In contrast to the part mass, the effects of the adjustment variables on the part dimensions are not reproduced.

#### 3.1.2. Use Case 2: Hexagonal Shaped Parts

**Figure 10:**
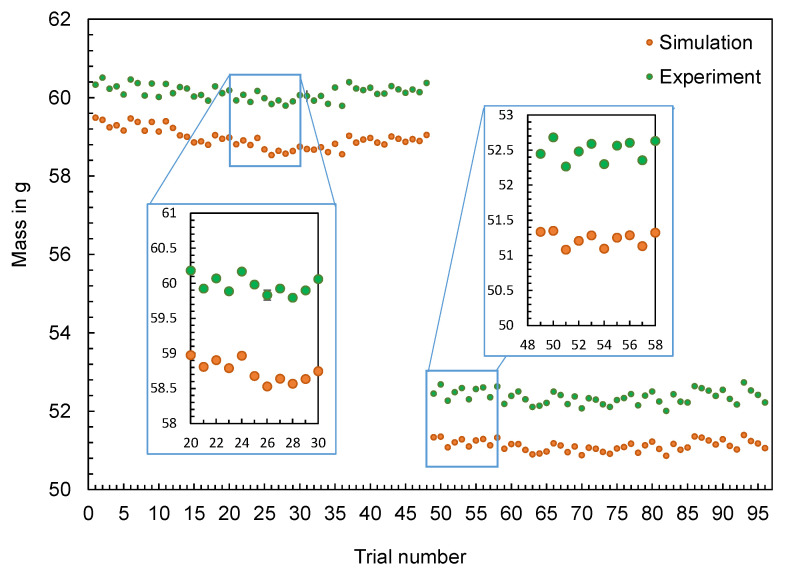
Computed and measured masses of the hexagonal shaped parts with (**left**) and without inner ring (**right**).

**Figure 11:**
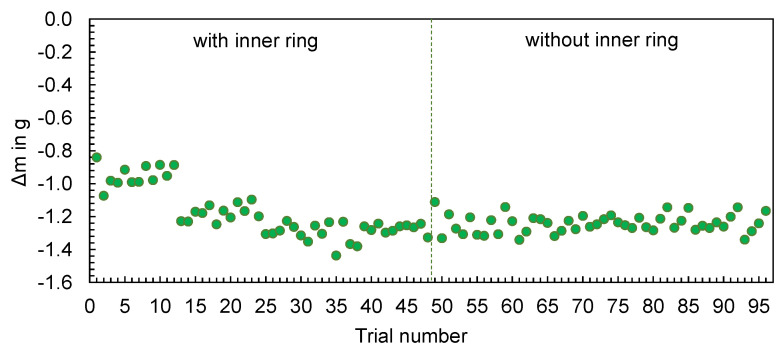
Deviation of the computed and mean measured masses of the hexagonal shaped parts.

The experimentally measured and computed masses of the hexagonal shaped parts are compared in Figure 10. The results confirm the findings generated for the flat bar: The changes in the machine setting parameters and their effect on the part mass (increase or decrease) are qualitatively reproduced by the simulation, but not quantitatively. The differences ∆m, calculated according to Equation (1), show an almost uniform offset over the complete series of tests (cf. Figure 11). Only at the beginning of the tests, a smaller difference in the values can be observed.

**Figure 12:**
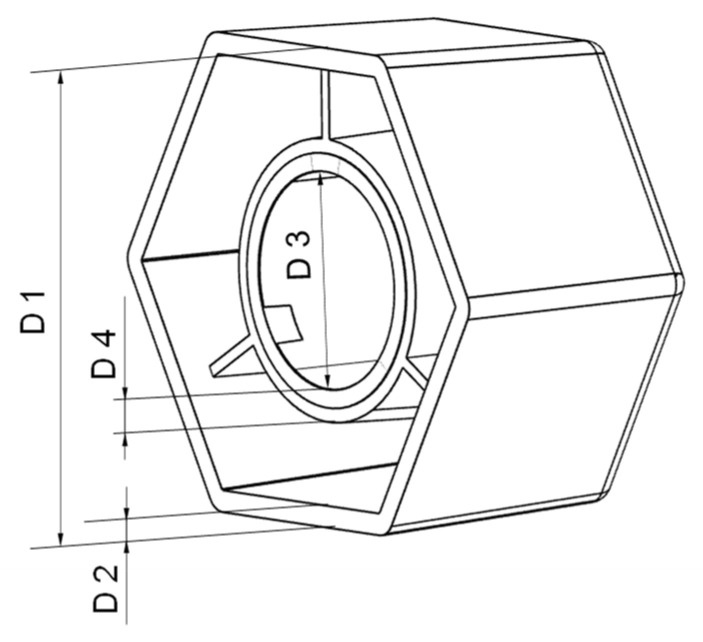
Dimensions (D) of the hexagonal shaped part for comparing simulation and experiment.

**Figure 13:**
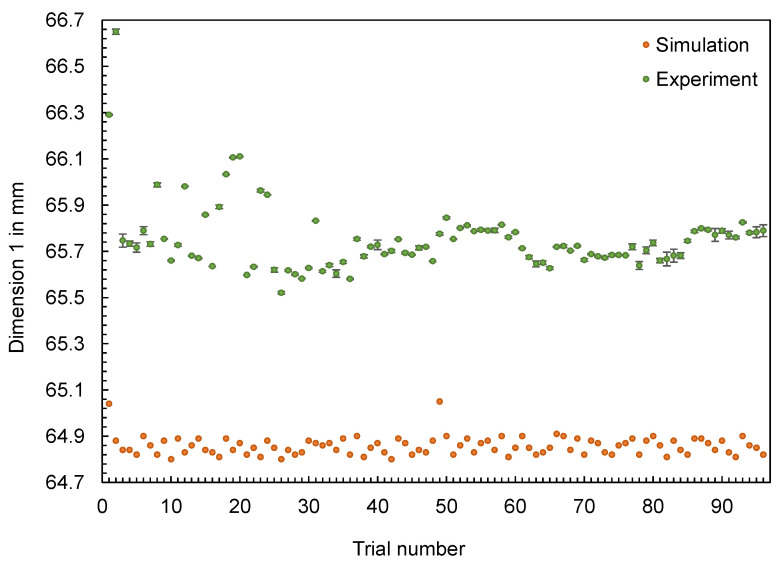
Comparison of simulation and experiment: Dimension 1 of the hexagonal shaped parts.

**Figure 14:**
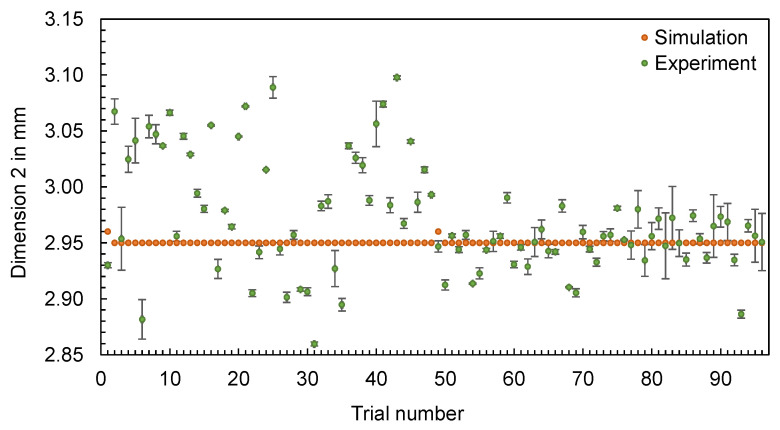
Comparison of simulation and experiment: Dimension 2 of the hexagonal shaped parts.

**Figure 15:**
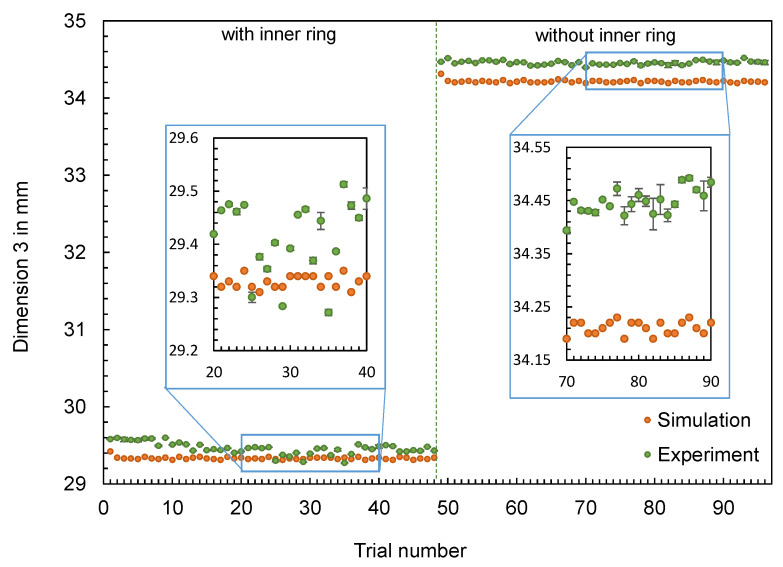
Comparison of simulation and experiment: Dimension 3 of the hexagonal shaped parts.

**Figure 16:**
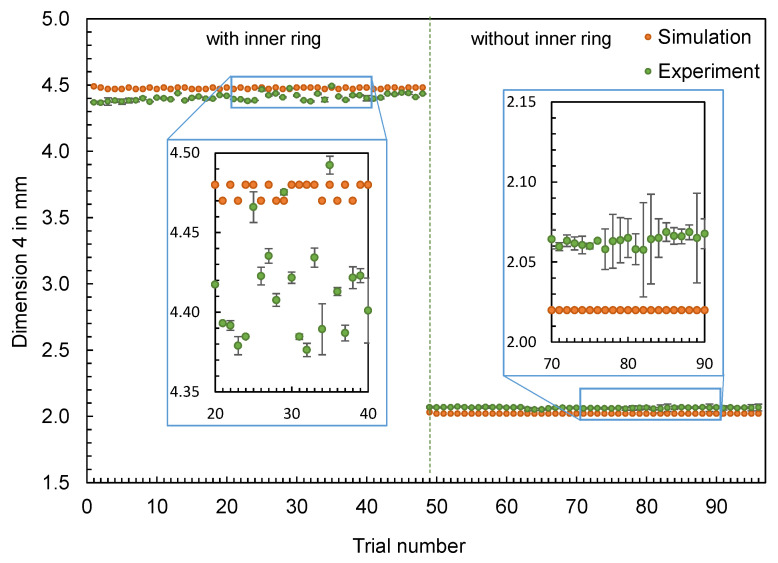
Comparison of simulation and experiment: Dimension 4 of the hexagonal shaped parts.

In addition to the part mass, the calculated and measured dimensions of the hexagonal parts are compared below. Four different dimensions were selected for this purpose (cf. Figure 12). The dimensions measured with the image-based measuring system and the values calculated by the simulation are shown in Figure 13, Figure 14, Figure 15 and Figure 16.

**Figure 17:**
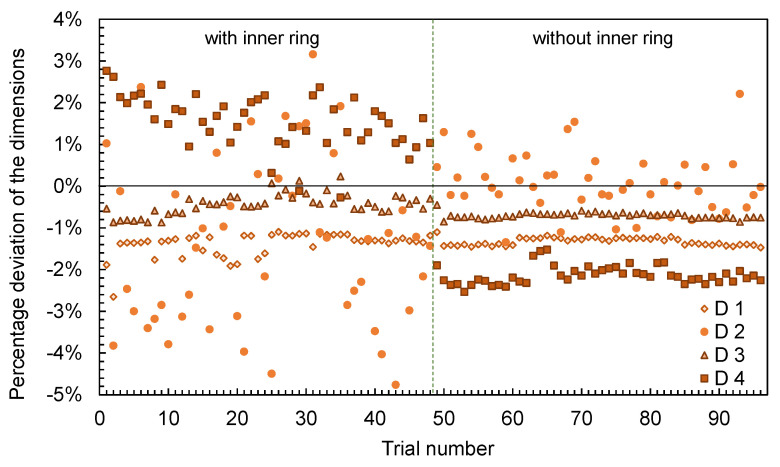
Percentage deviations between experimentally measured and calculated dimensions of the hexagonal parts.

The results also confirm the findings already generated for the flat bar: The effects of the setting parameters on the dimensions of the parts cannot be reproduced by the simulation. No obvious pattern can be identified in the partly very divergent results. To compare the results for the different dimensions, the differences ∆d were calculated according to Equation (2). Since a deviation between the simulated and measured dimensions is more significant for larger dimensions (such as D1), the percentage deviation was also calculated, with ∆d divided by the mean value of the measured dimensions. Based on the percentage deviations in Figure 17, it can be seen that some of the deviations are highly scattered (like D2, with an inner ring) and others show an almost constant offset (like D3, without an inner ring).

#### 3.1.3. Discussion of the Comparisons of the Calculated and Measured Part Characteristics (Use Case 1 and 2)

The comparisons of the part characteristics show that the simulation is able to represent the effect of operating point changes on the part mass, although in most cases there is an offset between calculated and measured part masses. When it comes to the calculated part thicknesses or dimensions, the scenario is different: The simulation is not able to represent changes in the operating points on the dimensions of the parts. Partly, the simulation calculates identical values for the part thickness despite variations in the setting parameters. Thus, it was shown that the part characteristics measured in the experiment did not match the simulated ones.

Potential causes for the partly significant deviations might be the following:

- *Lack of representation of plasticization and disturbance variables in the simulation:* The simulation software is not capable of modeling the plasticization of the material and only maps the process once the melt has entered the hot runner or mold. As already shown in [37], the back pressure and the screw rotational speed can influence the melt properties and consequently the part weight. A neglect of these machine settings in the simulation can therefore be one of the causes that result in a deviation between the calculation results and the experimental data. In addition to plasticizing, disturbance variables and their effects on the process are not simulated. Initial approaches to the integration of disturbance variables in the simulation have already been presented in [38]. The integration of such approaches in commercial simulation programs does not yet exist. In summary, the deviation between simulation and experiment depends on the extent to which the plasticization and disturbance variables in the experiment influence the material properties and the process and the degree to which these properties and process states deviate from the properties and states assumed in the simulation.
- *Influence of the specific behavior of the injection molding machine during the experiment, which cannot be reproduced by the simulation:* The specific behavior of the injection molding machine, which results in a specific process dynamic, can differ from the process behavior assumed in simulation. To investigate the machine-specific behavior and its influence on the process, additional studies were carried out, which are already published in [39]. It was shown that the injection molding machine can have a specific behavior depending on its type and operating point. To compare the extent to which this behavior of the machine deviates from the calculated process behavior, the calculated and measured time series of the process parameters are compared in Section 3.2.
- *Deviation of the characterized material properties used for simulation from the material properties during processing:* The pvT behavior of polymers is one of the most important factors with an influence on the shrinkage and warpage of the final products [40]. To check how well the material data used for numerical simulation fits to the actual material properties, the material used was analyzed. The results are presented in Section 3.3.
- *Influence of simulation parameters for discretizing the part geometry and for solving the equations on the calculation result:* The mesh and solver parameter can have an influence on the simulation result. An analysis of the extent of this influence and a comparison to experimental data are presented in Section 3.4.

In the following section, the results of the investigations for analyzing the potential causes are presented.

### 3.2. Comparison of the Calculated and Measured Time Series

In the experiment, both the material- and the machine-specific behavior can be responsible for the fact that adjusted machine settings are not directly realized as assumed in the simulation. To compare the actual process inertia with the “ideal process” assumed in simulation, the correspondences of the calculated and measured time series of the process parameter flow rate, injection pressure, and cavity pressure were examined. This involved comparing the progression of the time series at different process settings. Due to the large number of process settings investigated, the results of two selected operating points using the flat bar are presented below. For this, one operating point with a high expected viscosity (resulting from low cylinder temperatures, a low mold temperature and a low flow rate) and one with a low expected viscosity (resulting from high cylinder temperatures, a high mold temperature and a high flow rate) was specifically selected (see Table 3).

**Figure 18:**
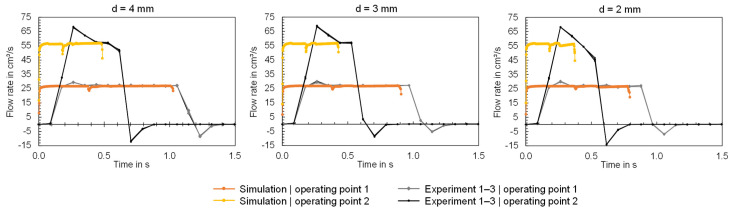
Comparison of the measured and calculated time series of the flow rate when using the flat bars with thicknesses of 4, 3 and 2 mm (f.l.t.r.).

The calculated and measured time series of the flow rates (Figure 18) demonstrate that in the simulation an immediate realization of the set flow rate is assumed. However, in the experiment, the machine shows a certain inertia, which means that the flow rate resulting from the screw stroke was realized with a time delay and exhibited typical transient behavior. Investigations into the machine-specific behavior of different injection molding machines and the influence on the time series of the process parameters have already been presented in [39]. The time series also show that the correspondence between simulation and experiment depends on the respective machine setting (here: flow rate). At higher flow rates, the machine overshoots the set flow rate (as presented in [39]) to a greater extent, which means that the experimental data deviates more strongly from the calculated data at high flow rates. In addition, at the end of the injection phase, a negative flow rate can be detected because of the decompression of the melt in the screw antechamber and an accompanying backward movement of the screw as a result of the switchover from a high injection pressure to a lower packing pressure. Both the inertia and the transient behavior during injection cause the measured and calculated durations of the injection phases to vary.

**Figure 19:**
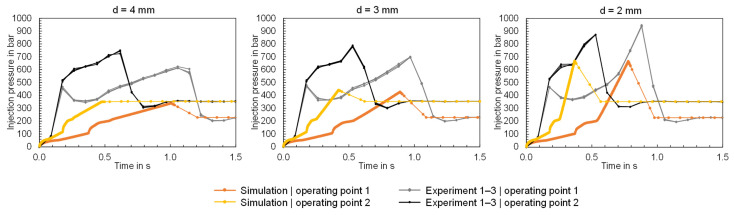
Comparison of the measured and calculated time series of the injection pressure when using the flat bars with thicknesses of 4, 3 and 2 mm (f.l.t.r.).

The comparison of the calculated and measured injection pressures is shown in Figure 19. The injection pressure recorded by the machine was measured via the hydraulics, which can result in differences between the actual injection pressure and the recorded injection pressure as a result of hydraulic pressure losses as well as pressure loss to melt flow in the cylinder and nozzle tip. This can lead to the simulation calculation deviating from the experimental measurement results because the simulation does not include corresponding effects.

**Figure 20:**
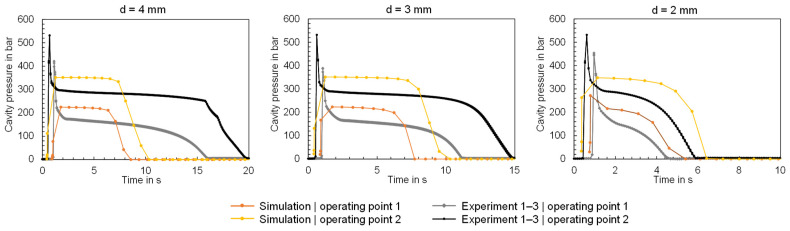
Comparison of the measured and calculated time series of the cavity pressure when using the flat bars with thicknesses of 4, 3 and 2 mm (f.l.t.r.).

The measured and calculated cavity pressures are compared in Figure 20. The cavity pressures reveal that the cavity pressure in the injection phase is underestimated but overestimated in the packing pressure phase. The latter is due to the factors of hydraulic pressure losses, the friction of the screw movement in the barrel, and melt flow pressure drop through the nozzle tip, which result in the effective packing pressure in the experiment being lower than the set packing pressure. Since these factors are not included in simulation, the packing pressure is overestimated. In addition, the calculated duration for which the packing pressure is still effective in the cavity deviates significantly from the measurement results. Both the neglect of the dependence of viscosity on pressure and the effect of mold deformation can lead to deviations between the measured and calculated cavity pressure profiles [41]. In addition, the thermal conductivity has an influence on the time to freeze of the material and consequently on the progression of the cavity pressure. Since the thermal conductivity depends on the temperature and the pressure, the use of a single value (which is normally used for process simulation) can lead to simulation inaccuracies [42].

The comparisons clearly show that both the actual material- and the machine-specific behavior are responsible for the fact that the conditions assumed in the simulation do not correspond to the physical experiment. At this point it must be emphasized that the findings obtained are valid exclusively for the production scenario investigated. Due to the complexity and diversity of injection molds, the variety of materials and machines as well as the multitude of operating point settings, a method that is able to calculate the correspondence between simulation and experiment in a data-driven manner is considered useful. A corresponding method, that evaluates the operating point-dependent similarity including the time series of the process parameters and also the part characteristics has already been defined in [43].

### 3.3. Comparison of the Actual Material Data and the Material Data Used for Simulation

To validate the material data used for simulation, the polyamide Ultramid B3S was measured using the high-pressure capillary rheometer (HCR) “Rheograph 25” provided by the company Göttfert Werkstoff-Prüfmaschinen GmbH (Buchen, Germany). Subsequently the pvT and viscosity data were compared with those provided by BASF and stored in the Moldflow database. Both in industry and in research, it is well established that standard measurement methods such as HCR are used to model the rheological behavior and the pvT properties of polymers. However, more process-realistic pvT data can be obtained if process-like conditions are achieved during the measurement process [44,45]. Nevertheless, standard measurement methods were used to be able to compare the input data of the simulation and the actual material properties using the same measurement procedure.

**Figure 21:**
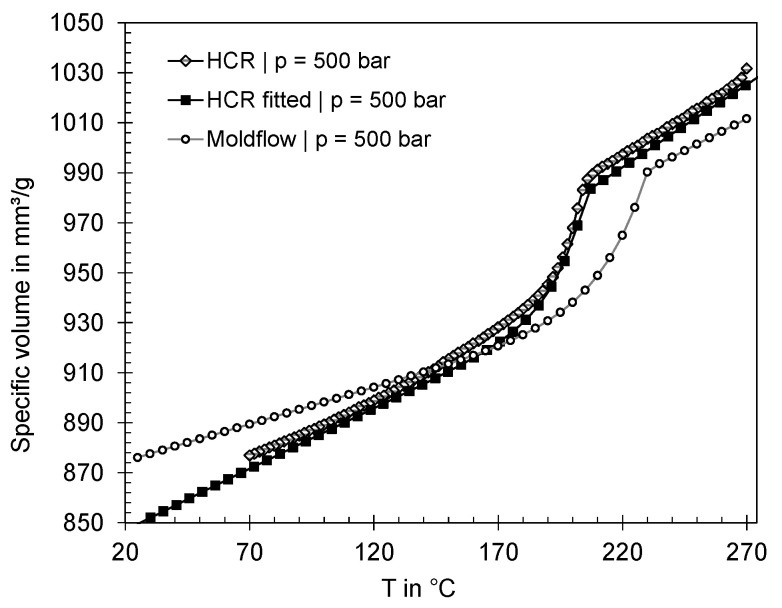
Comparison of the pvT data measured with the HCR and the 2-Domain Tait model fitted based on HCR data and those provided by Moldflow.

Figure 21 shows the pvT properties of the materials assessed with the HCR (measured with a capillary length of 25 mm, capillary diameter of 2 mm, and a cooling rate of 10 K/min), the fitted 2-Domain Tait model [18] based on HCR data (fitted with the Software Autodesk Moldflow Data Fitting 2024 [46]) and the 2-Domain Tait model provided by the Moldflow database. For the sake of clarity, only the pvT properties at a pressure of 500 bar are visualized. The data show that the transition ranges differ significantly, with a difference in transition temperature of approximately 20 °C. When comparing the data, it must be noted that the measuring method itself and the cooling rates have a significant influence on the position of the transition range between melt and solid. Different cooling rates can result in a shift of the transition temperature [47]. In particular, since it is not known at which cooling rates the generic data stored in Moldflow were measured, the difference in transition range may result from the measurement method. Nevertheless, not only a shift in the transition range can be observed, but also significant differences in the specific volume depending on temperature, which is shown by the fact that the specific volume of the measured material is lower below 140 °C and higher above 140 °C than the specific volume of the model stored in Moldflow.

**Figure 22:**
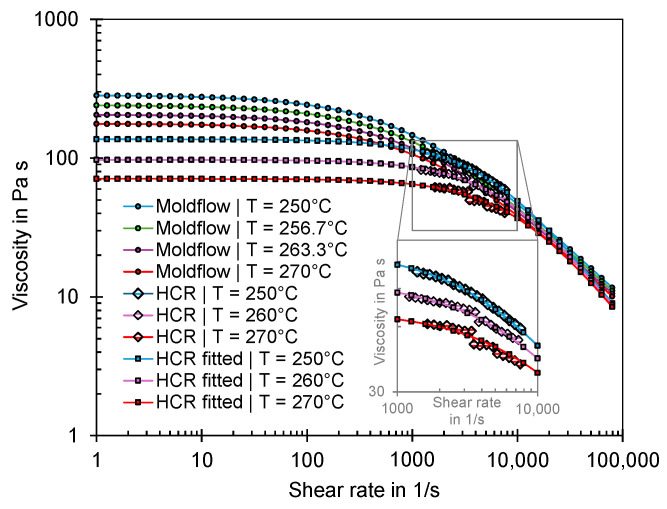
Comparison of the viscosities measured with the HCR and the Cross-WLF models fitted based on the HCR data and those provided by Moldflow.

In addition to the pvT properties, the viscosities at different temperatures were measured using the HCR (with a capillary length of 20 mm and capillary diameter of 0.5 mm). The measurement data were subsequently used to model the Cross-WLF model [17] using Autodesk Moldflow Data Fitting Software [46]. The fitted Cross-WLF model and the viscosity model provided by Moldflow is shown in Figure 22. The data show that the viscosities of the material processed during the experiments are lower than the viscosities described by the Cross-WLF model, which is stored in Moldflow.

In addition, the melt mass flow rate (MFR) was analyzed by using the measurement system “Meltflixer 2000” provided by the company Thermo Haake (Karlsruhe, Germany). The measurement was carried out at 275 °C (equivalent to the material measurement stored in Moldflow) and was repeated ten times. The measured MFR of 183 g/10 min (with a standard deviation of 2.7 g/10 min) also differs from the MFR of 170.6 g/10 min from the simulation.

**Figure 23:**
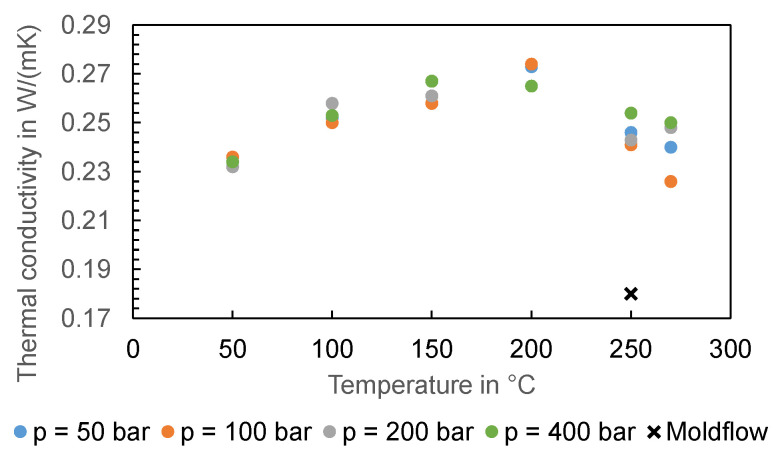
Comparison of the thermal conductivity measured and those provided by Moldflow.

To verify the thermal conductivity data in Moldflow, the thermal conductivity of the material was measured as a function of temperature and pressure according to ASTM D 5930 [48] using the HCR. The measurement results are shown in Figure 23. Moldflow provides a thermal conductivity of 0.18 W/(m K) at 250 °C, which deviates significantly from the thermal conductivity of 0.33 W/(m K) [49], which is provided by the material supplier.

**Figure 24:**
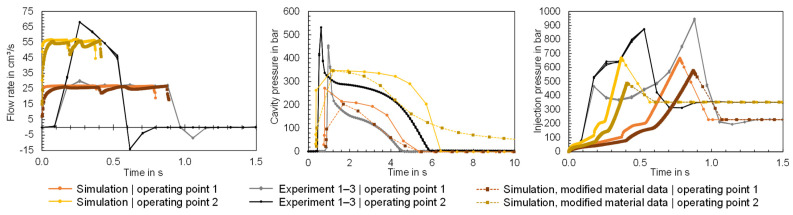
Comparison of the time series of the flow rate, cavity pressure and injection pressure measured in the experiment with the time series calculated in the simulation using the material data card from Moldflow and the material data fitted.

The comparisons show that the input data of the simulation can deviate from the actual properties and also the material information provided by the material supplier. Potential causes for the differences between the material data are material fluctuations, recipe changes or differences in the measurement procedures. The extent to which deviations between the material properties specified in the software and the properties of the material processed in the experiment can lead to a simulation error will be analyzed below. For this purpose, simulations were conducted using a modified material data card. The material data card of Ultramid B3S was used as a reference and the Cross-WLF model and the 2-domain Tait model were adjusted on the basis of the pvT and viscosity data measured using the HCR. In addition, the MFR was adjusted, and the thermal conductivity was added as a function of temperature. The modified material data card was used to compute the time series flow rate, injection pressure and cavity pressure for the flat bar geometry with a thickness of 4 mm. The time series measured in the experiment are compared in Figure 24 with the time series calculated via simulation. Both the simulation results using the material data stored in Moldflow (Figure 24 “Simulation”) and those calculated using the modified material data (Figure 24 “Simulation, modified material data”) are visualized.

The time series illustrate the influence of the material data on the computational result. For the flow rate, it can be observed that the length of the injection phase varies, with the newly calculated material data leading to a longer injection phase, which is more similar to the experimental data. The cavity pressure shows a high level of agreement with the measured cavity pressure, particularly at operating point 1 after the switchover. At operating point 2, however, the cavity pressure is effective for much longer than measured in the experiment. The pressure peaks of the cavity pressure during the injection phase on the other hand cannot be mapped. This results from the fact that the injection pressure is significantly underestimated despite the adjusted material data. On the one hand, the results demonstrate the influence of the material data on the simulation result and, on the other hand, that the material data are not solely responsible for the simulation error. The flow rate profile in particular suggests that the machine-specific behavior, which is not mapped by the simulation, leads to a simulation error. Further studies should focus in particular on the integration of the machine-specific behavior in the simulation.

### 3.4. Influence of the Mesh Density on the Simulation Result Using the Flat Bar as an Example

**Figure 25:**
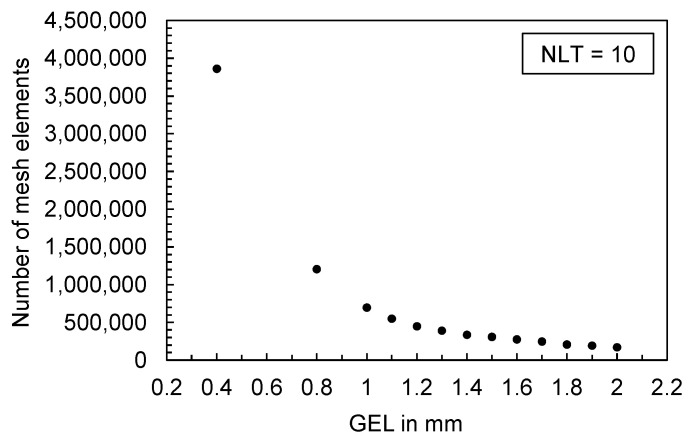
Number of mesh elements depending on the global edge length (GEL), flat bar with a thickness of 2 mm.

**Figure 26:**
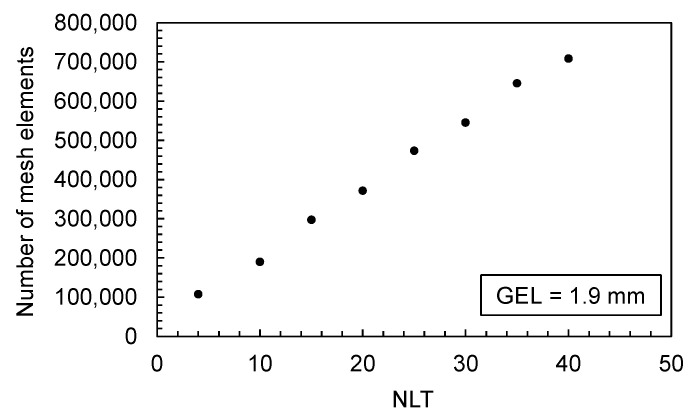
Number of mesh elements depending on the number of layers across the thickness (NLT), flat bar with a thickness of 2 mm.

The mesh density for a 3D mesh depends on the global edge length (GEL) and the number of layers across thickness (NLT). The following figures illustrate the number of mesh elements as a function of the global edge length (Figure 25) and the number of layers across the thickness (Figure 26) for the flat bar with a thickness of 2 mm as an example.

To investigate the influence of the mesh on the simulation result, the computed masses, the part thicknesses, and the computed time series are shown in the following depending on different mesh parameters. In addition, the simulation results are compared with the data measured in the process.

#### 3.4.1. Influence of the GEL and NLT on the Computed Time Series of the Process Parameters and Comparison with Experimentally Measured Data

**Figure 27:**
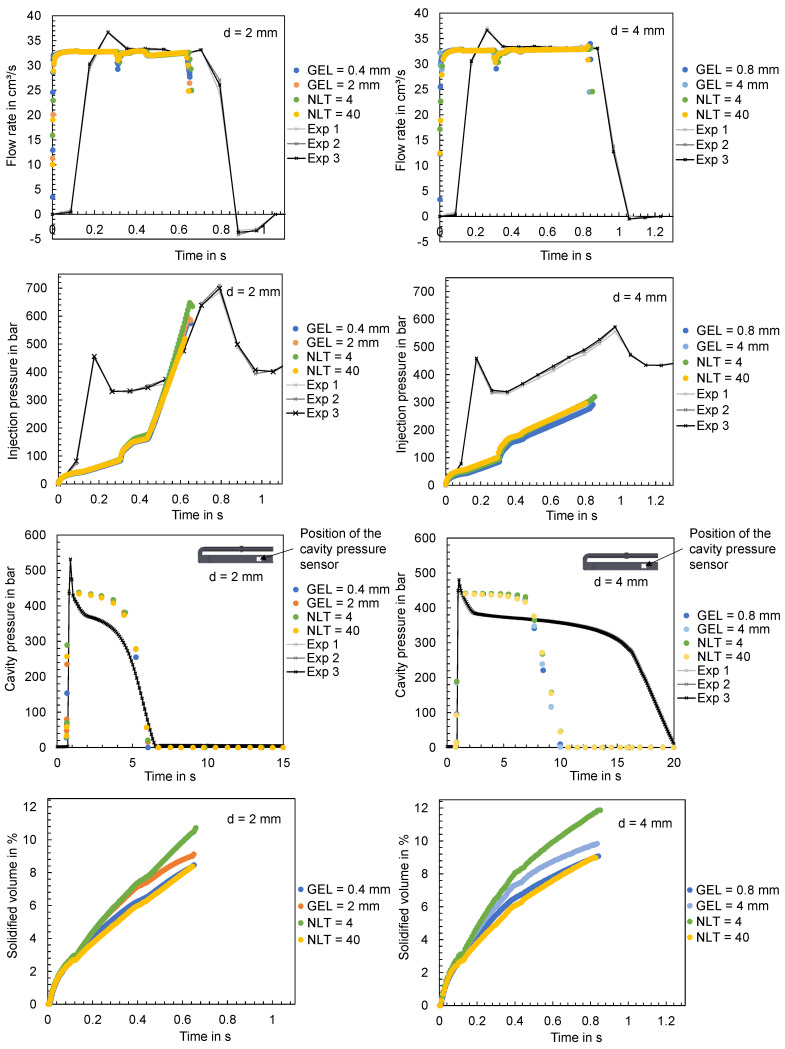
Comparison of the time series of the flow rates, injection pressures and cavity pressures measured in the experiment and the computed time series under variation in the mesh parameters; left: flat bar with d = 2 mm, right: flat bar with d = 4 mm.

For the evaluation of the influence of the GEL on the calculated time series of the process parameters, the flat bar with the settings from trial number 26 and 90 (shown in Table A2) were used as an example. Figure 27 shows the results of the flat bars with a thickness of 2 mm and 4 mm. Since the computed time series partly show high similarities, the results of the minimum and maximum varied GEL as well as NLT are visualized. The mesh parameters and the resulting number of mesh elements are shown in Table A5.

When comparing the experimental data with the simulation results, it is obvious that the influence of the mesh parameters is negligible compared to the difference between the computed and the in-process measured time series. The offset of the flow rate is especially noticeable. Viscoelastic effects and melt viscosity changes [50], but also the control and hydraulic of the injection molding machine, which are not taken into account in the simulation calculations, may be responsible for such a time delay. In addition to the flow rate, the calculated injection pressure as well as the cavity pressure show significant deviations from the data measured during the experiments.

The only parameter that shows significant deviations when varying the mesh parameters is the solidified volume. The time series show a decrease when the GEL is decreased, respectively, and when the NLT is increased (which results in a finer mesh). Whether the mesh density has an influence on the molded part mass will be analyzed below.

#### 3.4.2. Influence of the GEL and NLT on the Computed Mass Using the Flat Bar as an Example

**Figure 28:**
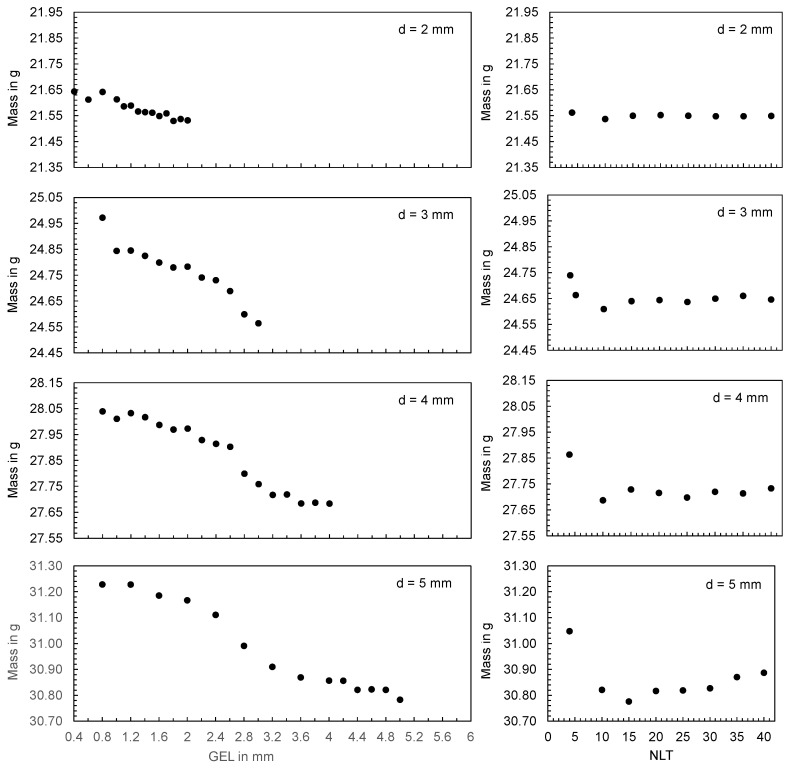
Computed mass at the end of the cycle for different flat bar thicknesses when varying the global edge length with 10 layers across the thickness (**left**) and varying the number of layers across the thickness with a global edge length of GEL = d − 0.2 mm (**right**).

Figure 28 shows the calculated mass at the end of the cycle as a function of the global edge length (left) and as a function of the number of layers across the thickness (right) for the different flat bar thicknesses. When varying the GEL, a constant value was chosen for the NLT (NLT = 10) and when varying the NLT, the GEL was chosen as a function of the flat bar thickness d (GEL = d − 0.2 mm).

Based on the plots, the following results can be described: In the simulation, both the GEL and the NLT and the associated mesh density have an influence on the part mass calculated. However, there is a contradictory relationship; by reducing the GEL and increasing the NLT, the mesh becomes finer, i.e., the number of mesh elements increases (cf. Figure 25 and Figure 26). When the mesh density is increased because of the reduction in the GEL, an increase in the calculated part mass can be observed. In contrast, a decrease in the calculated part mass can be observed when the mesh density is increased as a consequence of the increase in the NLT (especially at low NLT). Thereby, the calculated part mass seems to approach a lower limit range when the NLT is increased. In the case of the flat bar with a thickness of 5 mm, an increase in the part mass can again be seen at high NLT.

**Figure 29:**
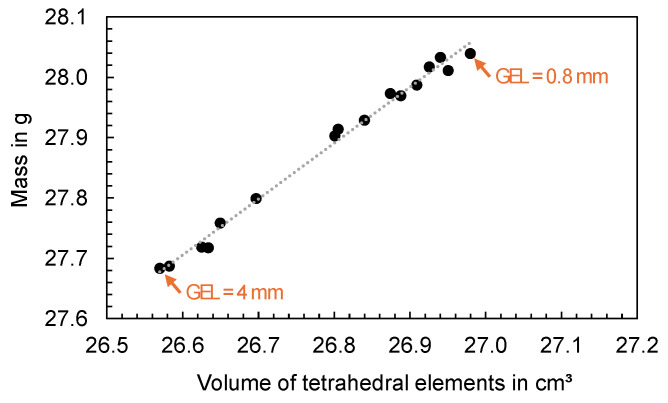
Computed mass as a function of the volume meshed with tetrahedral elements.

To analyze the influence of the faceting on the mass calculated, the volume of the tetrahedral elements was compared with the calculated mass (ct. Figure 29). A linear correlation was found which suggests that the volume of the geometry is insufficiently meshed when using high GEL, resulting in a reduction in the calculated mass.

**Figure 30:**
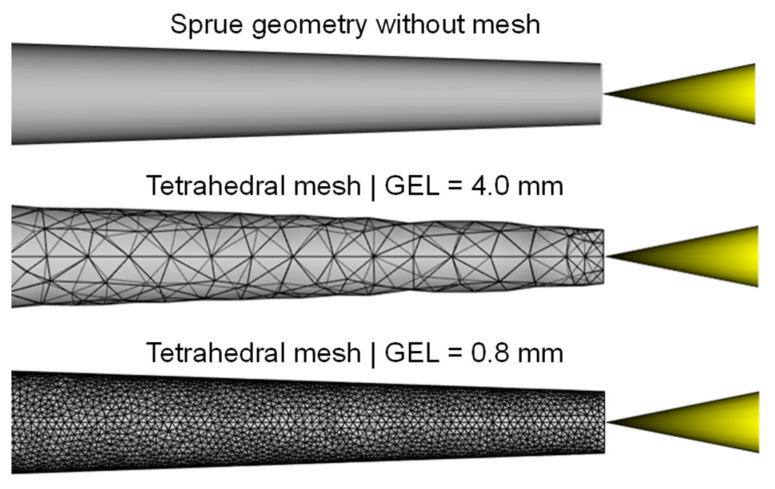
Comparison of the non-meshed sprue geometry and the tetrahedral meshes with a GEL of 4 mm and 0.8 mm.

Particularly in the areas of circular shapes, a deviation between the volume of the tetrahedral elements and the volume of the input geometry can be observed (ct. Figure 30). The higher the GEL, the smaller the volume of the tetrahedral elements, which means that the facets of the input geometry (especially in circular regions) are not correctly represented. This geometry discretization error has an influence on the calculation of the mass and consequently on the correspondence between the calculated and measured mass.

**Figure 31:**
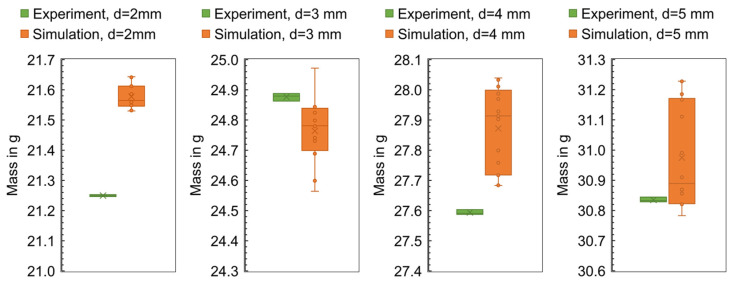
Comparison of the computed and measured masses at the end of the cycle for different flat bar thicknesses.

For an evaluation of the mesh density at which the simulation shows the best correspondence with the experiment, the part masses are compared for one operating point in Figure 31. From the boxplots, it can be seen that the simulation shows a wide variance when the mesh density is changed, with the ranges of values of the computed and measured part masses showing an intersection only for the flat bar thickness of 3 mm and 5 mm.

As a conclusion, the mesh density, which results from the GEL and the NLT, has a significant influence on the computation of the part mass. Thus, the mesh density can be responsible for the fact that the computed and measured part masses deviate from each other.

#### 3.4.3. Influence of the GEL and NLT on the Computed Part Dimensions

**Figure 32:**
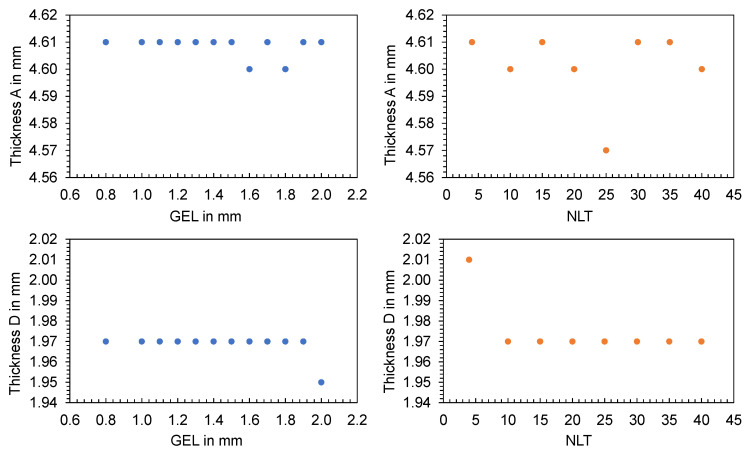
Thickness A and D of the flat bar with a thickness of 2 mm depending on the GEL (blue) and NLT (orange).

To evaluate the influence of the mesh on shrinkage and consequently on individual part dimensions, the 2 mm flat bar was meshed with different global edge lengths in the range from 0.8 mm to 2 mm. After performing the cooling, filling, and warpage analysis, the thicknesses were measured in the simulation software using the distance from the measuring points A and D to the respective opposite nodal points (cf. Figure 3). The calculated thicknesses are shown in Figure 32 as a function of GEL (left) and NLT (right) as well as measuring point A (top) and measuring point D (bottom).

**Figure 33:**
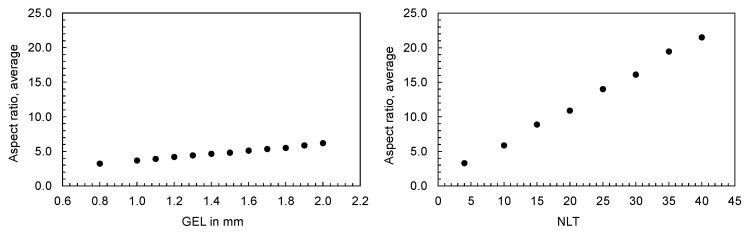
Resulting average aspect ratio when varying the GEL with constant NLT = 10 (**left**) and when varying the NLT with constant GEL = 1.9 mm (**right**).

The changes in thickness are not clearly dependent on the GEL or the NLT, but rather random. The average aspect ratio, which is the ratio of the longest side of an element to the height perpendicular to that side, was determined to check the quality of the mesh. The results, shown in Figure 33, reveal a smaller influence of the GEL on the aspect ratio compared to variations in the NLT. High aspect ratios and local mesh inhomogeneities can lead to simulation errors. Consequently, the choice of meshing parameters has an influence on the thickness and should be carefully selected before simulation and comparison with experimental data.

#### 3.4.4. Influence of the Solver Parameters on the Computed Time Series

Depending on the mesh type used, different assumptions for the solver are defined in the software. The assumptions, such as the Navier–Stokes equations for a 3D mesh, have already been presented. In addition, the maximum volumetric fill per time step in the filling phase (MVFTS) and the number of intermediate results in the filling phase (IRF) can be varied as solver parameters.

The investigation of the solver parameters focused exclusively on the filling phase since the packing pressure phase is pressure-controlled and no deviations between the calculated time series are to be expected there. The various studies and the corresponding solver settings can be found in Table 4.

**Figure 34:**
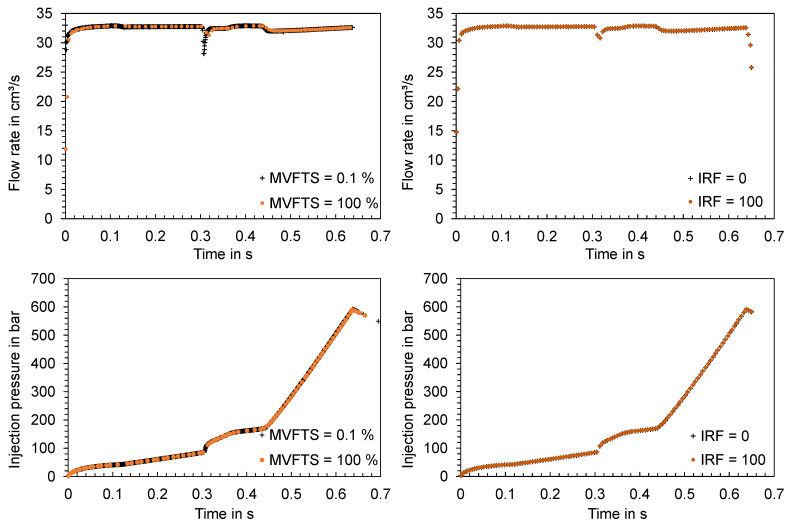
Time series of the flow rate and injection pressure in the injection phase as a function of the solver parameters MVFTS and IRF.

The time series computed for the injection flow rates and the injection pressures are visualized in Figure 34. It becomes apparent that the different solver settings have an influence on the number of calculated time steps and that local minima cannot be mapped at a higher volumetric filling per time step (MVFTS = 100%) in contrast to a low volumetric filling per time step (MVFTS = 0.1%). In the time course, the series show no discernible differences.

**Figure 35:**
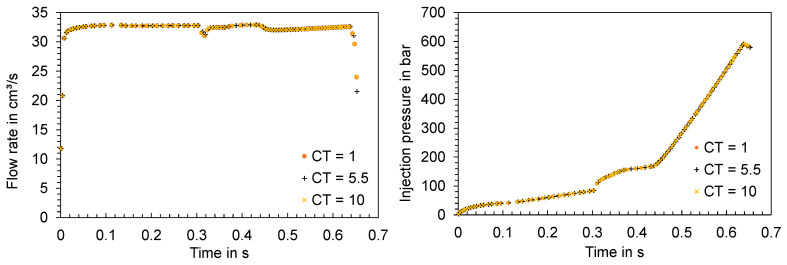
Time series of the flow rate and injection pressure in the injection phase as a function of the solver parameter CT.

In addition to the solver parameters presented, the convergence tolerance (CT) was varied using the settings of the study “MVFTS = 100%” (listed in Table 4) and changing the CT values. A total of three simulations were run with CT values of 1, 5.5, and 10. Comparable to the variation in the solver parameters MVFTS and IRF, no significant differences in the computed time series can be identified when varying the CT (ct. Figure 35).

Overall, the investigation of solver parameters reveals no significant impact on the simulation results, thus ruling out the possibility that these parameters play a significant role in the disparity between the simulation and experimental outcomes.

### 3.5. Simulation of the Material and Machine-Specific Behavior

**Figure 36:**
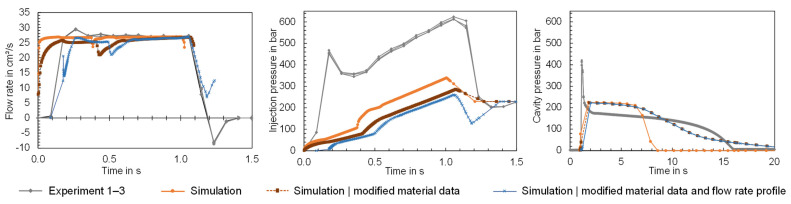
Comparison of the time series of the process parameters measured in the experiment (“Experiment”) with the time series computed in the simulation using different input data for simulation (here: flat bar with a thickness of 4 mm and operating point 1).

**Figure 37:**
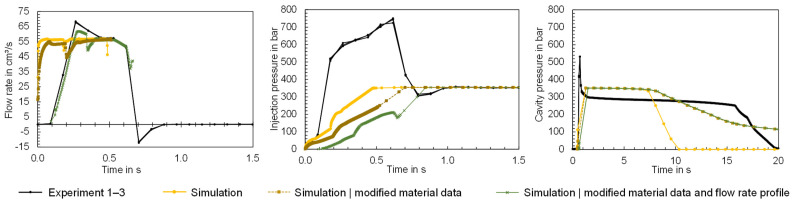
Comparison of the time series of the process parameters measured in the experiment (“Experiment”) with the time series computed in the simulation using different input data for simulation (here: flat bar with a thickness of 4 mm and operating point 2).

According to Section 3.2, Section 3.3 and Section 3.4, the material and machine-specific behavior and their representation in the simulation have a major influence on the time series of the process parameters and thus the correspondence between simulation and experimental results. To analyze the ability of the simulation to reproduce the dynamics of the experiment, additional simulations were carried out for the flat bar with a thickness of 4 mm considering the operating points shown in Table 3. For the simulation, both the new material data card developed in Section 3.3. and the machine-specific behavior identified in Section 3.2. were considered. To incorporate the machine-specific behavior in simulation, instead of the scalar value for the nominal flow rate, the measured flow rate profile was specified as input for the simulation. The absolute ram speed profile was specified for the configuration of the filling control in the simulation, whereby the screw stroke of 65.5 mm was configured as the starting ram position, which corresponds to the dosing volume of 32.12 cm^3^ measured in the experiment. To compare the influence of the modified material data card and the machine-specific behavior on the simulation result, Figure 36 and Figure 37 show the computed time series of the process parameters when using the material data provided by Moldflow and a constant flow rate (“Simulation”), using the modified material data card and a constant flow rate (“Simulation|modified material data”) and using the modified material data card and a flow rate profile (“Simulation|modified material data and flow rate profile”) as an input for the simulation. When comparing the simulation results with the experimental data (“Experiment”) in the case of operating point 1 (Figure 36) and operating point 2 (Figure 37), the following can be concluded:

By predefining the flow rate profile, the simulation is able to take the movement of the screw into account, although there are still differences between the measured and computed flow rates. Since the simulation calculates the decompression of the melt in the screw antechamber [51] and the flow rate profile of the melt entering the sprue [52], this explains a deviation in the time series, especially at the beginning of the injection phase. Compared to the calculated flow rates, where a constant flow rate was specified in the simulation, the difference between simulation and experiment was significantly reduced by including machine-specific behavior. As a result of the dynamic behavior of the machine, the computed injection pressure and cavity pressure increase with a delay. The dynamic behavior influences the course of the injection pressure, but does not particularly influence the course of the cavity pressure (compared to the time series “Simulation|modified material data”). The reasons for the persistent difference between the computed and measured pressures have already been discussed in Section 3.2.

**Figure 38:**
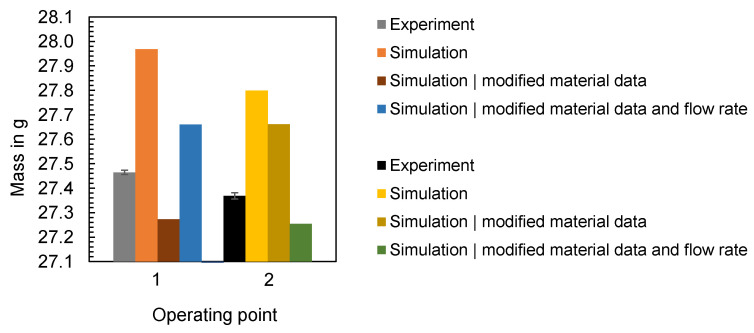
Comparison of the part mass measured in the experiment (“Experiment”) with the mass computed in the simulation using different input data for simulation.

To compare not only the process parameters, but also to obtain an initial impression of the influence of the material and the dynamic behavior on the computational result of the part characteristic mass, Figure 38 is used. Both factors, material and machine, have a significant influence on the calculated mass, whereby the difference to the measured quality characteristic was reduced. In future studies, a detailed comparison between simulation and experiment should be carried out, in which both the actual material behavior and the dynamic behavior of the injection molding machine are integrated in the simulation. In this context, the determination of the rheological properties using an inline measurement method (as presented in [53,54]) and the description of the machine-specific behavior using a so-called machine fingerprint should be of interest.

## 4. Conclusions

Calculation results, generated with the commercially available simulation software Moldflow Insight Ultimate, were compared with experimental data to analyze the extent to which simulation can be used as a substitute for an experiment. Considering different Use Cases involving different mold geometries, the quality characteristics of the parts and the time series of the process parameters were compared. Subsequently, potential causes for the deviation between simulation and experiment were discussed and analyzed. In summary, the following results were obtained:

- When comparing the computed and experimentally measured characteristics of different injection molded parts, it was found that the congruence depends strongly on the respective characteristics under consideration. The comparison of the part masses showed a congruence of the values in some cases and a certain offset in others. Depending on the geometry considered, a different offset was found. In particular, the effect of changes in the operating point on the part mass was well reproduced by the simulation, although the magnitude of the change varied in comparison with the experiment. This was different when comparing various thicknesses of the flat bars or dimensions of the hexagonal shaped parts. In the experiment, changes in dimensions and thicknesses were identified, whereas the simulation showed partly constant or slightly scattered values. Thus, the effects of operating point variations during the experiments on the measured dimensions of the parts cannot be adequately computed by using the simulation model applied.
- In addition to the part characteristics, the time series of the process parameters injection pressure, flow rate, and cavity pressure measured in the experiment and computed by the simulation were compared. It was found that the simulation partly shows significant deviations from the time series measured during the process. The measured flow rates showed a time delay and an over-shooting in contrast to the time series computed. As a result of the offset, the injection times also differed. In addition, the injection pressure and cavity pressure showed significant deviations from the calculated time series. The potential causes are the negligence of the machine-specific behavior, the viscoelastic behavior, or disturbance variables occurring in the process. Further investigations with regard to the machine-specific behavior were carried out and are published in [39]. It was shown that the process, in particular the process dynamics, is essentially influenced by the specific and operating point dependent behavior of the injection molding machine. Neglecting these dynamics in the simulation consequently means that the calculated time series of the process parameters in particular show insufficient correspondence with the data measured in the experiment.
- The comparison of the pvT, viscosity, thermal conductivity, and MFR data measured with the material data used as an input for the simulation showed that deviations from the actual material properties can exist when using the material data from the database. This can be due to batch variations, changes in material production or differences in measurement procedure, among other things. Consequently, the material data itself can be responsible for a discrepancy between simulation and experiment.
- When analyzing the influence of the mesh parameters on the simulation result, it was shown that the computation of the part mass depends significantly on the selected GEL and the resulting volume of the tetrahedral elements. Likewise, a dependence on the NLT was shown, especially at low values. In contrast, when analyzing the influence of the mesh parameters on individual dimensions, no unambiguous dependence on the mesh parameters was shown. The partly random variations in the thickness as a result of mesh parameter variations suggest that the quality of the mesh in particular influences the quality of the dimension calculation. The influence of the mesh quality on the computed part characteristics should be investigated in more detail in further studies. For this purpose, GEL-NLT-pairs should be used that result in similar aspect ratios to investigate the influence of the mesh fineness on the computational result while maintaining the same mesh quality.
- A variation in the mesh parameters has no significant influence on the time series of the process parameters, especially compared to the experimentally measured data, which showed a large deviation from them. Consequently, the mesh parameters cannot be responsible for the discrepancy of the process parameters.
- The solver parameters have an influence on the number of calculated data points, but no influence on the course of the time series. Here it is recommended to choose the solver parameters in favor of the calculation time.

The studies demonstrate that the similarity between simulation and experiment depends on the operating point, the production scenario and its dynamics, and also the validity of the material data. Due to the limitations of the simulation, e.g., mapping the dynamic behavior of the injection molding machine, training machine learning models based on simulation data alone are not yet adequate to represent the actual injection molding process. A disregard of the operating point-, machine-, and material-dependent similarity between simulation and experiment may be a reason why simulation-pretrained models (like investigated in [26]) sometimes work well and sometimes not at all when transferring to the actual process. To be able to use simulations as a substitute for experiments or to pre-train models with simulation data, it is recommended to use a method that evaluates in a data-driven manner which simulation has a high correspondence with the experiment. A corresponding approach has already been developed and is presented in [43].

## Figures and Tables

**Table 1:** Description of the injection molding machines.

	A	B
Test specimen (assembled mold)	Flat bar	Hexagonal shaped parts
Clamping force, kN	500	1100
Max. injection flow rate, cm^3^/s	66	136
Screw diameter, mm	25	30
Nozzle diameter, mm	4	3
Max. injection pressure, bar	2500	2000
Calculated stroke volume, cm^3^	59	85
Effective screw length (length/diameter), -	24	20
Drive	hydromechanic	

**Table 2:** Parameters used for the configuration of the simulation studies.

Parameter	Flat Bar	Hexagonal Shaped Part
Mesh type	3D Tetrahedra	3D Tetrahedra
Global edge length, mm	1	0.75
Number of layers	16	20
Solver	Coupled 3D	Coupled 3D
Solution type	Stokes	Stokes
Viscosity model	Cross-WLF	Cross-WLF
Hot runner	no	yes
Gate diameter at injection locations	3 mm	2 mm
Mold dimensions	300 mm × 140 mm × 160 mm	-
Velocity/pressure switch-over control	By % volume filled	By % volume filled
Switch-over, Percentage volume of the part	98%	99%
Maximum % volume to fill per time step	1%	1%
Maximum iterations per time step (filling and packing)	50	50
Maximum time step packing	0.088 s	0.088 s

**Table 3:** Selection of operating points used for the comparison of the calculated and measured time series.

Operating Point	T_c_, °C	T_m_, °C	Q_inj_, cm^3^/s	p_pack_, Bar
1	250	50	27	229
2	270	80	57	354

**Table 4:** Solver parameter settings.

	Study	MVFTS = 0.1%	MVFTS = 100%	IRF = 0	IRF = 100
Solver Parameter					
Filling parameters					
Maximum % volume to fill per time step (MVFTS)		0.1	100	1	1
Maximum iterations per time step		50	50	50	50
Convergence tolerance (scaling factor)		1	1	1	1
Packing parameters					
Maximum time step		0.088 s	0.088 s	0.088 s	0.088 s
Maximum iterations per time step		50	50	50	50
Convergence tolerance (scaling factor)		1	1	1	1
Intermediate results					
Number of intermediate results in filling phase (IRF)		20	20	0	100
Number of intermediate results in packing phase		20	20	20	20
Number of intermediate results in cooling phase		20	20	20	20

## Data Availability

The data presented in this study are available on request from the corresponding author.

## Appendix A

**Table A1:** Heating zone temperatures for manufacturing the flat bars and hexagonal shaped parts.

T_c_, °C	Heating Zones, °C
250	250; 250; 245; 240; 235; 50
270	270; 270; 265; 260; 255; 50

**Table A2:** Experimental design for the configuration of the simulation and the experiment for producing the flat bars.

Trial Number	T_c_, °C	T_m_, °C	Q_inj_, cm^3^/s	p_pack_, Bar
1 + j	250	50	49	496
2 + j	250	50	33	443
3 + j	250	50	57	354
4 + j	250	50	50	302
5 + j	250	50	27	229
6 + j	250	50	37	334
7 + j	250	50	44	266
8 + j	250	50	23	406
9 + j	250	80	49	496
10 + j	250	80	33	443
11 + j	250	80	57	354
12 + j	250	80	50	302
13 + j	250	80	27	229
14 + j	250	80	37	334
15 + j	250	80	44	266
16 + j	250	80	23	406
17 + j	270	80	49	496
18 + j	270	80	33	443
19 + j	270	80	57	354
20 + j	270	80	50	302
21 + j	270	80	27	229
22 + j	270	80	37	334
23 + j	270	80	44	266
24 + j	270	80	23	406
25 + j	270	50	49	496
26 + j	270	50	33	443
27 + j	270	50	57	354
28 + j	270	50	50	302
29 + j	270	50	27	229
30 + j	270	50	37	334
31 + j	270	50	44	266
32 + j	270	50	23	406

**Table A3:** Variable j depending on part thickness di, addendum to Table A2.

Thickness d_i_ in mm	j
4	0
3	32
2	64
5	96

**Table A4:** Experimental design for the configuration of the simulation and the experiment for producing the hexagonal shaped parts.

Trial Number	Core	T_c_, °C	T_m_, °C	Q_inj_, cm^3^/s	p_pack_, Bar
1	0	250	50	53	377
2	0	250	50	24	445
3	0	250	50	48	353
4	0	250	50	28	361
5	0	250	50	54	302
6	0	250	50	32	494
7	0	250	50	21	403
8	0	250	50	44	291
9	0	250	50	38	437
10	0	250	50	26	255
11	0	250	50	45	469
12	0	250	50	35	319
13	0	250	70	21	412
14	0	250	70	50	472
15	0	250	70	53	348
16	0	250	70	27	329
17	0	250	70	32	269
18	0	250	70	35	486
19	0	250	70	24	362
20	0	250	70	30	419
21	0	250	70	47	304
22	0	250	70	40	376
23	0	250	70	42	281
24	0	250	70	55	449
25	0	270	70	26	382
26	0	270	70	39	262
27	0	270	70	34	362
28	0	270	70	42	301
29	0	270	70	22	331
30	0	270	70	34	466
31	0	270	70	53	427
32	0	270	70	56	412
33	0	270	70	30	446
34	0	270	70	48	346
35	0	270	70	18	497
36	0	270	70	44	291
37	0	270	50	57	496
38	0	270	50	28	287
39	0	270	50	35	377
40	0	270	50	44	427
41	0	270	50	47	314
42	0	270	50	39	261
43	0	270	50	49	468
44	0	270	50	52	414
45	0	270	50	25	299
46	0	270	50	22	357
47	0	270	50	33	336
48	0	270	50	20	453
49	1	270	50	46	373
50	1	270	50	23	482
51	1	270	50	33	291
52	1	270	50	39	384
53	1	270	50	51	441
54	1	270	50	37	303
55	1	270	50	27	414
56	1	270	50	20	432
57	1	270	50	42	329
58	1	270	50	56	472
59	1	270	50	29	264
60	1	270	50	48	351
61	1	270	70	22	476
62	1	270	70	19	361
63	1	270	70	56	285
64	1	270	70	52	303
65	1	270	70	44	339
66	1	270	70	32	497
67	1	270	70	36	454
68	1	270	70	47	326
69	1	270	70	44	434
70	1	270	70	26	268
71	1	270	70	39	411
72	1	270	70	30	392
73	1	250	70	38	317
74	1	250	70	46	290
75	1	250	70	48	379
76	1	250	70	37	400
77	1	250	70	41	459
78	1	250	70	26	302
79	1	250	70	31	428
80	1	250	70	24	481
81	1	250	70	55	370
82	1	250	70	52	259
83	1	250	70	28	448
84	1	250	70	20	343
85	1	250	50	38	297
86	1	250	50	30	473
87	1	250	50	51	453
88	1	250	50	33	408
89	1	250	50	23	343
90	1	250	50	54	425
91	1	250	50	48	320
92	1	250	50	35	269
93	1	250	50	25	496
94	1	250	50	21	395
95	1	250	50	42	357
96	1	250	50	47	286

**Table A5:** Mesh parameters, in addition to Figure 27. The colors of the dots correspond to the colors of the data points in Figure 27.

Study	GEL in mm	NLT	Mesh Elements		Study	GEL in mm	NLT	Mesh Elements	
GEL = 0.4 mm	0.4	10	3 857 649	•	GEL = 0.8 mm	0.8	10	1 301 581	•
GEL = 2 mm	2	10	169 822	•	GEL = 4 mm	4	10	47 834	•
NLT = 4	1.9	4	107 424	•	NLT = 4	3.8	4	22 334	•
NLT = 40	1.9	40	708 698	•	NLT = 40	3.8	40	196 992	•
